# Recent Advances in Carbon-Based Single-Atom Catalysts for Electrochemical Oxygen Reduction to Hydrogen Peroxide in Acidic Media

**DOI:** 10.3390/nano14100835

**Published:** 2024-05-09

**Authors:** Hao Yin, Ronglan Pan, Manman Zou, Xin Ge, Changxuan Shi, Jili Yuan, Caijuan Huang, Haibo Xie

**Affiliations:** Department of Polymer Materials and Engineering, College of Materials and Metallurgy, Guizhou University, Huaxi District, Guiyang 550025, China; 19118329592@163.com (H.Y.); panronglan1929@163.com (R.P.); 18083698461@163.com (M.Z.); 15934764490@163.com (X.G.); scx15865116613@163.com (C.S.)

**Keywords:** carbon-based single-atom catalysts, oxygen reduction reaction, hydrogen peroxide, electrocatalysis, acidic media

## Abstract

Electrochemical oxygen reduction reaction (ORR) via the 2e^−^ pathway in an acidic media shows great techno-economic potential for the production of hydrogen peroxide. Currently, carbon-based single-atom catalysts (C-SACs) have attracted extensive attention due to their tunable electronic structures, low cost, and sufficient stability in acidic media. This review summarizes recent advances in metal centers and their coordination environment in C-SACs for 2e^−^-ORR. Firstly, the reaction mechanism of 2e^−^-ORR on the active sites of C-SACs is systematically presented. Secondly, the structural regulation strategies for the active sites of 2e^−^-ORR are further summarized, including the metal active center, its species and configurations of nitrogen coordination or heteroatom coordination, and their near functional groups or substitute groups, which would provide available and proper ideas for developing superior acidic 2e^−^-ORR electrocatalysts of C-SACs. Finally, we propose the current challenges and future opportunities regarding the acidic 2e^−^-ORR pathway on C-SACs, which will eventually accelerate the development of the distributed H_2_O_2_ electrosynthesis process.

## 1. Introduction

Hydrogen peroxide (H_2_O_2_) is considered a sustainable chemical due to its eco-friendly and efficient characteristics, playing a significant role in promoting the development of the green chemical industry [1,2,3]. At present, the production of H_2_O_2_ mainly adopts the traditional energy-intensive anthraquinone method. However, the anthraquinone synthesis method usually requires large-scale infrastructure and generates a large number of waste liquids [3], which poses transportation costs and safety problems in the transportation and storage process of H_2_O_2_. Although H_2_O_2_ could be directly synthesized from hydrogen (H_2_) and oxygen (O_2_), the mixture of H_2_ and O_2_ is easily explosive, which limits its application [1,4]. Therefore, the development of an instant, efficient, safe, and stable H_2_O_2_ production method has become the research focus of scientific researchers.

The electrochemical pathway provides an environmentally friendly, efficient, and safe production effective way for the production of H_2_O_2_. There are usually two possible reaction pathways for H_2_O_2_ electrosynthesis. One is through the two-electron oxygen reduction reaction pathway (2e^−^-ORR), and the other is via the two-electron water oxidation reaction pathway (2e^−^-WOR). However, 2e^−^-WOR is not suitable for large-scale application because of the easy further oxidization of H_2_O_2_ [5,6]. The 2e^−^-ORR reaction process can stably produce high purity and high concentration H_2_O_2_, with only water as the by-product [4,7,8]. Although ORR can produce H_2_O_2_ in acidic, alkaline, and neutral media, the production of H_2_O_2_ in acidic conditions has many advantages over alkaline or neutral conditions. First, H_2_O_2_ is more chemically stable in acidic media than in neutral and alkaline media, which can effectively inhibit its self-decomposition [4]. Second, H_2_O_2_ electrosynthesis in acidic media can be used in the mature proton exchange membrane (PEM) device with excellent ionic conductivity and excellent stability [9]. Third, H_2_O_2_ in acid media enables its application in more areas of industrial processes [7]. However, H_2_O_2_ is thermodynamically unfavorable in the ORR [9], and reasonable design and preparation of electrocatalysts with low overpotential, high selectivity, and stable performance towards a 2e^−^-ORR pathway is a precondition for H_2_O_2_ production in acidic media.

Currently, multiple materials have been investigated as promising 2e^−^-ORR electrocatalysts in acidic solutions [3,10,11], including noble metals, transition metals, and carbon-based materials. Although noble metal-based materials have outstanding ORR activity and selectivity in acidic media, their application is limited by their scarcity and toxicity [12,13]. In recent years, carbon-based materials have been widely used for the H_2_O_2_ synthesis via the 2e^−^-ORR pathway due to their abundance, low cost, and ease of preparation compared to noble metal catalysts [14]. The active sites can be increased in the carbon skeleton through defect control [15,16], pore control [17], and heteroatom functionalization [18,19,20]. However, the 2e^−^-ORR performance of the carbon-based catalysts is still restricted by the high overpotential and low selectivity of H_2_O_2_ under acidic conditions. It is worth noting that transition metals with 3d orbital electrons have higher activity of oxygen reduction in acidic conditions due to their excellent affinity for O_2_ [21,22]. Therefore, carbon-based transition metals single-atom catalysts (C-SACs) formed by combining transition metals and carbon-based materials have attracted much attention due to their advantages of both the maximum atomic utilization efficiency and the adjustable structure of SACs, and the low cost of carbon-based materials [23,24,25,26,27]. Although many electrocatalysts for 2e^−^-ORR in acidic conditions have been widely reported and reviewed, there is still a lack of systematic elaboration of C-SACs for H_2_O_2_ electrosynthesis in acidic solution.

Herein, this review concentrates on the research progress of C-SACs for H_2_O_2_ electrosynthesis by 2e^−^-ORR in acidic electrolytes. Firstly, we summarize the fundamental principles of the oxygen reduction process in H_2_O_2_ electrosynthesis. Secondly, the structural regulation strategy, including the metal active center, its species and configurations of nitrogen coordination or heteroatom coordination, and their near functional groups or substitute groups, are proposed to boost the performance of C-SACs in 2e^−^-ORR. Finally, the current challenges and future opportunities of C-SACs for H_2_O_2_ electrosynthesis in acidic conditions are discussed, providing a new guideline for designing efficient catalysts in the promising field of electrocatalytic 2e^−^-ORR to produce H_2_O_2_.

## 2. Reaction Mechanism of the ORR to H_2_O_2_

**Figure 1:**
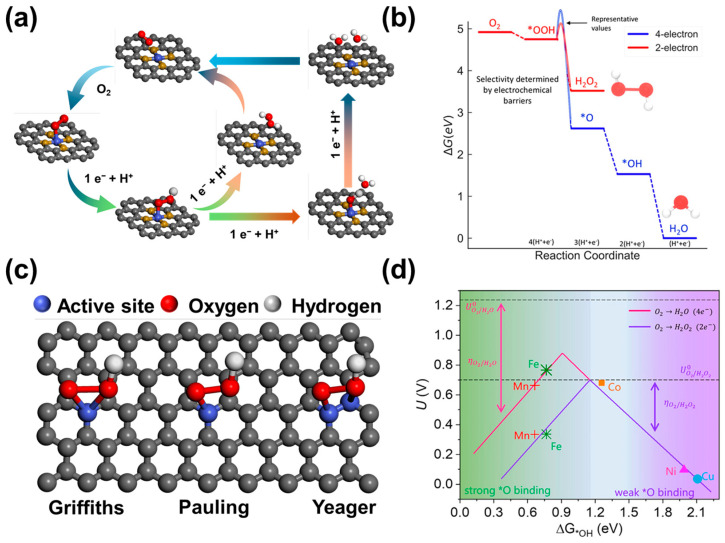
The reaction mechanism of 2e^−^-ORR by C-SACs in acidic media. (**a**) Schematic illustration of 2e^−^ and 4e^−^-ORR pathways. (**b**) Free energy diagram of 2e^−^ and 4e^−^-ORR pathways on Au (111) surface [9]. Copyright 2018, American Chemical Society. (**c**) Three adsorption modes of molecular O_2_ on catalytic active sites. (**d**) Effect of different d-band centers of metal atoms in M-N-C SACs (M = Mn, Fe, Co, Ni, Cu) on the generation of current H_2_O_2_ [39]. Copyright 2020, Elsevier.

In general, the ORR is a complex process involving multistep proton-coupled electron transfer, which can proceed through the competing 2e^−^ and 4e^−^ reaction paths (Figure 1a). The specific reaction paths in acidic media are as follows in Equations (1) and (2):

Four-electron pathway:

O_2_ + * + 4H⁺ + 4e^−^ → 2 H_2_O  E^0^ = 1.23 V vs. RHE (1)

Two-electron pathway:

O_2_ + * + 2H⁺ + 2e^−^ → H_2_O_2_  E^0^ = 0.70 V vs. RHE (2)

O_2_ + * + H^+^ + e^−^ → *OOH (3)

*OOH + H^+^ + e^−^ → H_2_O_2_ + * (4)

The “*” denotes the active center of the catalyst.

Typically, H_2_O_2_ is the final product for the 2e^−^-ORR pathways in an acidic solution, with *OOH being the only intermediate. On the other hand, the 4e^−^-ORR pathways usually lead to H_2_O generation, involving three reaction intermediates of *OOH, *O, and *OH, which are extensively studied for metal–air batteries [28,29] and fuel cells [30,31]. The ORR pathway can be further divided into dissociative pathway and associative pathway mechanisms according to whether O_2_ dissociates on the electrode surface [32]. Specifically, the dissociation of O-O bonds occurs when the O_2_ is adsorbed on the electrode surface, resulting in the reduction of *OOH, *O, and *OH. Meanwhile, the formation of O-O bonds is retained by the *OOH intermediate, which presents the association mechanism. There is a common feature with the *OOH intermediate in the two reaction mechanisms from the perspective of kinetic reaction pathways. It should be noted that the binding free energy of *OOH on the catalyst (∆G_*OOH_) should be suitable, protecting the O–O bond from catalysis [33]. Furthermore, the 4e^−^-ORR has a standard electrode potential corrected by the 2e^−^-ORR thermodynamically, thus the reduction process is more likely to proceed in the direction of H_2_O generation by the 4e^−^ORR pathway (Figure 1b). Therefore, the key issue for H_2_O_2_ electrosynthesis lies in improving the selectivity and activity of the 2e^−^-ORR.

The *OOH intermediate is a necessary step for the generation of H_2_O_2_ by 2e^−^-ORR, thus the key to improving the selectivity of 2e^−^-ORR is to keep the O-O bond [34]. The ORR pathway and products are mainly determined by the adsorption mode of the O_2_ molecule on the catalyst surface [35]. There are three different modes of adsorption for the O_2_ molecule on the metal surface: Pauling type, Griffith type, and Yeager type [8,36,37] (Figure 1c). The Griffith and Yeager type exhibit a side-on O_2_ adsorption, which can extend and break the O-O bond, and the resulting *OH and *O intermediates can be combined with the active site, producing large amounts of H_2_O_2_ via the 4e^−^-ORR pathway [4]. In contrast, the Pauling type displays the end-on adsorption, reducing the possibility of O-O bond cleavage and advancing selectivity for H_2_O_2_ production [36,38]. Thus, the selectivity of 2e^−^-ORR can be improved by optimizing the adsorption of O_2_ on the catalytic surface. Fortunately, the isolated active centers on C-SACs are conducive to the adsorption of Pauling type, having a broad application prospect in the field of H_2_O_2_ electrosynthesis by 2e^−^ ORR in acidic media.

In addition, whether the O-O bond breaks or not also depends on the strength of the *OOH binding to the surface of the catalytic center. Ideally, the binding strength of O_2_ on the active site should be sufficiently strong to facilitate the generation of *OOH while the adsorption of *OOH should be low enough to allow the desorption of H_2_O_2_ [4]. Based on the calculated hydrogen electrode (CHE) model proposed by Nørskov [4], the volcano plot of the relationship between the thermodynamic limit potential (U_L_) and the adsorption binding energy of *OOH on different metal surfaces can be obtained by the free adsorption energy (∆G_*OOH_) of *OOH (or *OH) on the metal surface (Figure 1d). Generally, the *OOH adsorption should be thermodynamically favorable at the peak of the volcano plot corresponding to the equilibrium potential (U = 0.7 V vs. RHE) in an ideal 2e^−^ORR catalyst. The formation of *O and *OH is facilitated by the strong binding of *OOH to catalysts on the left segment of the volcano, leading to the dominance of the 4e^−^ pathway. Moreover, the weak binding of the *OOH to the right segment of the volcano can enhance the selectivity of H_2_O_2_ for the positioned catalysts [39]. Hence, the electronic structure of the catalytic active center can achieve the appropriate *OOH adsorption energy by the regulation of metal active centers, nitrogen species, heteroatom doping, surface functional groups, and local microenvironments.

## 3. Carbon-Based Single Atom Catalysts

The ideal 2e^−^-ORR catalysts should have high activity, selectivity, and stability for electrochemical H_2_O_2_ production in acidic media [8,14]. Compared with noble metal and transition metal-based catalysts, C-SACs formed by the combination of transition metal and carbon-based materials have attracted extensive attention due to the abundance and tunable structural properties of carbon materials [40]. The main factor for the remarkable performance of C-SACs is attributed to the synergic effect between the metal center and the surrounding coordination atom [26,41,42]. This section presents the strategies of C-ASCs to achieve H_2_O_2_ production in acidic media, including the structural regulation of metal active centers, nitrogen species, heteroatom doping, surface functional groups, and local microenvironments.

### 3.1. Metal Active Center Regulation Strategy

**Figure 2:**
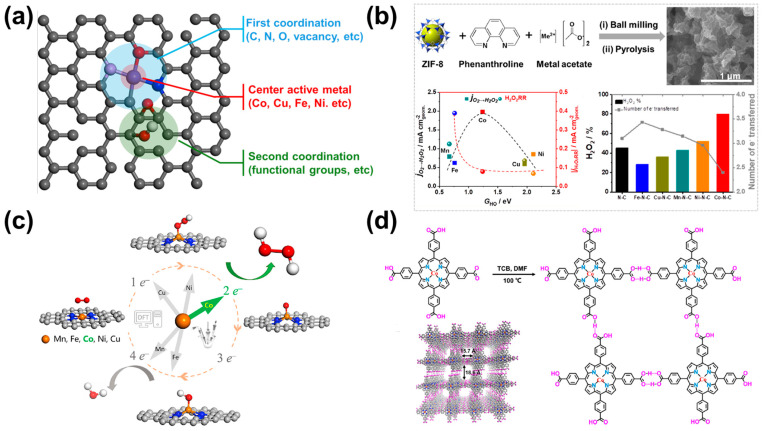
C-SACs for H_2_O_2_ production in the acidic condition. (**a**) Schematic illustration of SACs. (**b**) Scheme of synthesis of M-N-C SACs (M = Co, Ni, Fe, Cu, and Mn) and H_2_O_2_ selectivity and number of electrons at +0.1 V vs. RHE derived from RRDE data [40]. Copyright 2019, Elsevier. (**c**) Schematic of the ORR along the 2e^−^ or 4e^−^ pathway on transition metal SACs (M = Mn, Fe, Co, Ni, and Cu) anchored in N-doped graphene [39]. Copyright 2020, Elsevier. (**d**) Schematic synthetic procedure toward PFC-72-Co [22]. Copyright 2022, Springer Nature.

C-SACs based on transition metals typically feature individual metal centers dispersed and coordinated within a carbon-based matrix at the atomic level. The combination structure of C-SACs and naturally occurring metalloenzyme systems provides in-depth and comprehensive insights into the active sites involved in ORR. Similar to metalloenzymes, the catalytic efficiency of C-SACs can be affected by the metal active center, the atoms in the first coordination sphere, and the functional groups in the second coordination sphere (Figure 2a) [4]. The ORR selectivity of C-SACs majorly depends on the active center metal atoms. Thus, selecting a metal center atom is the most direct and effective means to improve the performance of the 2e^−^-ORR. Sun et al. prepared 3d metal of M-N-C (M = Mn, Fe, Co, Ni, Cu) SACs by pyrolysis after ball milling using ZIF-8 and 1,10-phenanthroline as carbon and nitrogen sources and transition metal as the metal source and studied the effect on the 2e^−^-ORR activity and selectivity [40]. Electrochemical tests and DFT calculations suggest that the Co-N-C catalyst has the most suitable *OH binding energy and the lowest H_2_O_2_ reduction reaction (H_2_O_2_RR) activity, thus showing the glorious ORR activity and the highest H_2_O_2_ selectivity (80% at 0.1 V vs. RHE) in the acidic condition. However, Fe-N-C and Mn-N-C catalysts with strong binding energy tend to generate H_2_O by the 4e^−^ ORR process, while Ni-N-C and Cu-N-C have low reaction activity due to their weak adsorption of Ni and Cu surfaces (Figure 2b). In another study, Gao et al. systematically studied the relationship between the structure and performance of the transition metal (Co, Mn, Fe, Ni, and Cu) SACs anchored on nitrogen-doped graphene by combining theoretical and experimental methods [39]. Particularly, the H_2_O_2_ production kinetic current over Co-NC could reach 1 mA cm^−2^_disk_ at 0.6 V vs. RHE in 0.1 M HClO_4_ with a faraday efficiency (FE) > 90%, and these performances could be maintained for 10 h without any decay (Figure 2c). Thus, the Co-NC is considered to be a promising electrocatalyst for the production of H_2_O_2_, even slightly outperforming state-of-the-art noble metal-based electrocatalysts in acidic media.

The metal macrocyclic compounds with a well-defined M-N_4_ structure (M = Co, Fe, Mn, Cu, etc.) provide open sites to bind with O_2_, but their development is still relatively insufficient [21,43,44]. It is necessary to promote their intrinsic catalytic activities further and enhance the surface exposition of the active sites [22]. In order to confirm the crucial role of metal centers, Zhao et al. screened 32 metalloporphyrin compounds with different metal centers by using high-throughput DFT calculations and confirmed that cobalt porphyrin exhibits the highest 2e^−^-ORR catalytic selectivity and activity [22]. Based on the results of the theoretical prediction, the authors developed a highly stable porphyrin cobalt with hydrogen-bonded organic frameworks (HOFs). The porphyrin-cobalt catalyst (PFC-72-Co) in 0.1 M HClO_4_ exhibited an onset potential at ~0.68 V vs. RHE, selectivity of H_2_O_2_ was over 90%, turnover frequency of 10.9 s^−1^ at 0.55 V, and stability of ~30 h (Figure 2d). These results show that the adsorption of Co in transition metals on *OOH intermediate is the most modest in M-N-C monoatomic catalysts or macrocyclic molecular catalysts, which is the most favorable for the synthesis of H_2_O_2_ by 2e^−^-ORR in the acidic condition. In subsequent studies, a large number of monoatomic catalysts with Co as the active center or template catalyst have appeared, showing excellent 2e^−^-ORR performance for the generation of H_2_O_2_ [24,26,43].

### 3.2. Effect of Nitrogen Coordination

**Figure 3:**
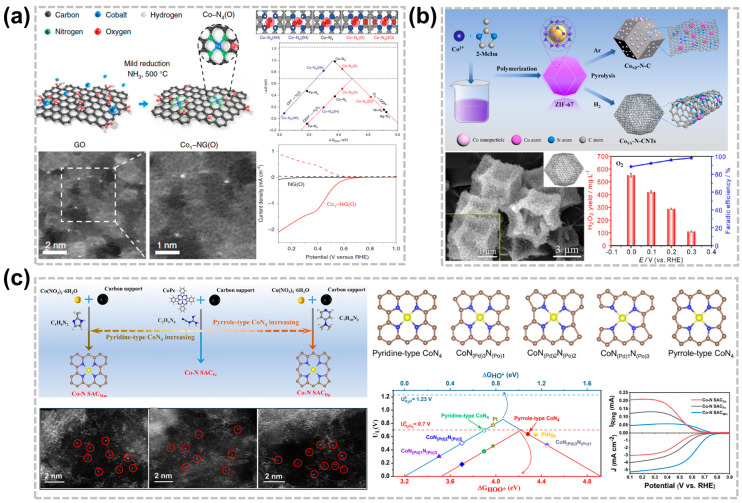
Effect of nitrogen coordination of C-SACs for acidic H_2_O_2_ production. (**a**) Schematic diagram of the synthesis of Co1-NG(O) and ORR activity volcano plot [47]. Copyright 2020, Springer Nature. (**b**) Schematic illustration for the preparation of CoSA-N-CNTs and CoNP-N-C catalysts [26]. Copyright 2022, Elsevier. (**c**) Effect of pyrrole type and pyridine type CoN_4_ structures on the catalytic performance of 2e^−^-ORR in acidic medium [2]. Copyright 2022 American Chemical Society.

The effective production of H_2_O_2_ in acidic media is significantly affected by the carbon substrate of C-SACs due to changes in core metal atoms and the surrounding coordination environment, including the coordination number, coordination groups, and coordination mode [34]. Altering the coordination environment shifts the 3d orbital central of the active center atom, significantly affecting the binding energy between the central metal atom and the *OOH and affecting the catalytic performance of the 2e^−^-ORR reaction [43]. Nitrogen group doping is an effective method to enhance the electrocatalytic activity of carbon materials for the 2e^−^-ORR. The N atoms can be well-doped into the carbon skeleton to form N-doped carbon materials with high stability and durability since the radii of N and C atoms are quite close to each other. Furthermore, the doping of the N atom allows the reaction to proceed via either a 2e^−^ or 4e^−^ ORR pathway [45,46]. Jung et al. designed and synthesized by first-principles calculations a Co-NG(O) that consists of an optimized Co-N_4_ moiety incorporated into nitrogen-doped graphene [47], which was synthesized by adsorbing metal atoms onto the surface of graphene oxide using impregnation, followed by a mild reduction in an NH_3_/Ar solution at 500 °C. DFT calculations indicate that the *OOH binding energy can be adjusted by adding electron-rich or electron-poor species to the Co-N_4_ site, significantly boosting the activity of the 2e^−^-ORR. In addition, the charge states of the Co atom are −0.21e^−^ and −0.35e^−^ in the coexistence of Co-N_4_ (2 H) and Co-N_4_ (4 H) while charge states are 0.05e^−^ and 0.10e^−^ in the presence of Co-N_4_ (O) and Co-N_4_ (2 O). Thus, the increase in the charge state of the Co atom and the decrease in the binding energy of *OOH is mainly attributed to the addition of electron-rich oxygen in the CoN_4_ site, resulting in the increase in ∆G_*OOH_. Experimental results show that Co_1_-NG(O) has higher onset potential and H_2_O_2_ selectivity in alkaline or acidic environments, which further proves that CoN_4_ is the 2e^−^-ORR active site (Figure 3a). 

Currently, the most effective method for preparing an M-N-C catalyst is the pyrolysis of metal-organic frameworks (MOFs), which can maintain an excellent porous structure with abundant active metal centers and heteroatomic dopants, resulting in high electrochemical performance [48,49]. However, the nanoparticles formed during the pyrolysis process will greatly reduce the selectivity of H_2_O_2_ electrosynthesis due to its dominance in the competitive reactions of the 4e^−^ pathway [50]. To avoid the formation of nanoparticles, Liu et al. design two types of Co-N-C active catalytic centers through the pyrolysis of ZIF-67 in a different atmosphere, which could be transformed effectively from 4e^−^ to 2e^−^ ORR by tuning the structural properties [26]. The fabricated CoSA-N-CNTs display extraordinary performance for a H_2_O_2_ production rate of 974 ± 25 mmol g_cat_^−1^ h^−1^ in acidic conditions along with an ultra-fast degradation performance of organic matter (Figure 3b). Similarly, Zhang et al. prepared the catalyst with a highly distributed Co atom anchored in porous N-doped carbon (p-Co-N-C) in ZIF-67 through the strategy of carbonization-alkalization-acidification and demonstrated its efficiency for H_2_O_2_ electrosynthesis in the acidic solution [51]. Although the Co C-SAC shows highly effective performance for the 2e^−^-ORR, the Co-N_4_ structure is usually presented as a highly active site for the 4e^−^ pathway in many previous studies [50,51,52,53]. Therefore, it is highly controversial that the structure-function connects the Co-N_4_ site structure and the 2e^−^ or 4e^−^ ORR pathway. The limited understanding is unfavorable for the design and preparation of highly active and selective catalysts for the 2e^−^-ORR in acidic media. Chen et al. systematically investigated the influence of different N atom types in the coordination structure of CoN_4_ on the catalytic performance of the ORR [2]. The results show that there are two unpaired electrons in the Co 3d orbital on the pyridine-type CoN4 structure, and the adsorption binding energy for the *OOH intermediate is relatively weaker, which is conducive to the desorption of the *OOH intermediate from the surface to produce H_2_O_2_. However, the Co 3d orbital does not contain unpaired electrons and has a strong binding force with *OOH after the pyridine-type CoN_4_ structure adsorbs the intermediate of *OOH, and the O-O bond is more likely to be broken, making the ORR more prone to the 4e^−^ process (Figure 3c). In addition, the true catalytic active sites of Fe-N-C electrocatalysts are still controversial. Hu et al. studied 13 different N-coordinated FeNxC configurations and their corresponding ORR activity [52]. Pyrrolic FeN_4_C shows the highest activity in acidic media, indicating that the coordinating pyrrolic N contributes to higher activity than that of pyridinic N.

### 3.3. Effect of Heteroatom Coordination

**Figure 4:**
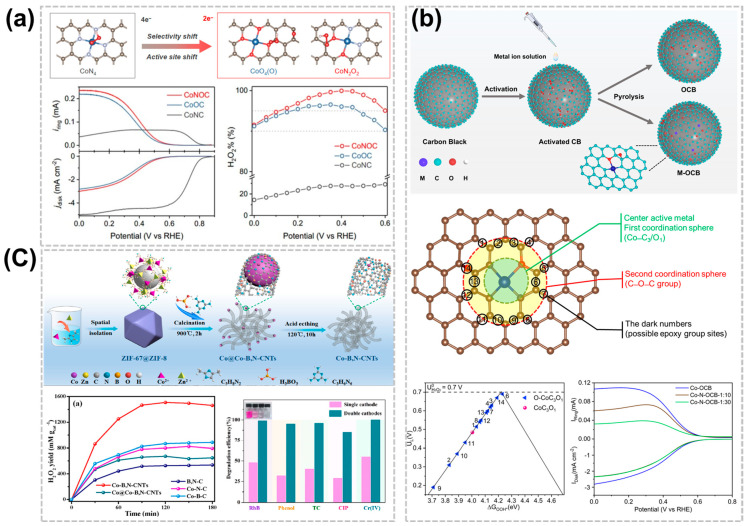
Effect of heteroatom coordination of C-SACs for acidic H_2_O_2_ production. (**a**) Optimized geometry structures of *OOH adsorption on CoNOC and its 2e^−^-ORR performance in acidic media [53]. Copyright 2021 American Chemical Society. (**b**) Schematic of metal OCB synthesis and theoretical prediction of Co-OCB at the molecular level [54]. Copyright 2023, Elsevier. (**c**) Scheme of the synthesis strategy for Co-B,N-CNTs and its time-dependent H_2_O_2_ production curves and the degradation efficiency of various pollutants [56]. Copyright 2023, Elsevier.

In addition to N-doping, the adsorption and catalytic properties of C-SACs for intermediate species *OOH can also be adjusted by heteroatom doping such as O, S, P, B, etc. [53,54,55]. Tang et al. reported the structure-property relationship of C-SACs catalysts via ORR in the acidic condition, for the first time showing that the molecular-level local structure synergistically affects the electrocatalytic performance [53]. The ORR selectivity of Co-SACs in commercial carbon black (CB) can be tailored from a 4e^−^ to a 2e^−^ pathway by changing the first (N or/and O coordination) and second (C-O-C groups) coordination spheres. Theoretical and experimental studies confirm that the unique selectivity change is attributed to a structure-dependent transfer of the active site from the central Co atom to the O-adjacent C atom. The as-designed CoNOC exhibits highly significant activity approaching the onset potential of ~0.57 V vs. RHE, selectivity of >95%, and 11 h stability for H_2_O_2_ synthesis in acidic media (Figure 4a). Similarly, Yuan et al. report that the activated commercial CB was used to anchor a single transition metal catalyst with first and second coordination sites [54]. As a result, Co-SACs exhibit extraordinary performance for ORR to produce H_2_O_2_, where a current density of around ~2.8 mA cm^−2^ at 0.1 V vs. RHE in rotating ring-disk electrodes and a high yield rate of 110.2 mmol g_metal_ ^−1^ h^−1^ at the potential of 0.3 V vs. RHE in a gas-diffusion-electrode (GDE) cell, with a high positive onset potential of 0.65 V vs. RHE and a >80% selectivity of H_2_O_2_ at the potential range from 0.25 to 0.65 V vs. RHE, respectively. The CO-OCB showed remarkable performance in the production of H_2_O_2_ in the 2e^−^-ORR in acid medium, with a current density of ~2.8 mA cm^−2^ at 0.1 V vs. RHE, a yield of 110.2 mmol g_metal_^−1^ h^−1^ at GDE cell, and a selectivity of >80% at 0.25-0.65 V vs. RHE. The theoretical calculations show that the catalytic center is located in the Co-C_3_/O_1_ radical of the adjacent epoxy group. Furthermore, the possible site of the epoxy group in the second coordination sphere was also studied (Figure 4b). Yang et al. proposed a nanotube reactor strategy via B, N-doped defective carbon nanotubes for the pursuit of high activity for the 2e^−^ ORR in acidic conditions [56]. The Co-B,N-CNTs produced a high yield of 1508 mmol L^−1^ g_cat_^−1^ of H_2_O_2_ with remarkable activity and selectivity via the 2e^−^ ORR pathway in HClO_4_ solution, ascribing to the Co-SACs with the uniformly dispersed and defect-rich active sites. In addition, the in-situ generation of H_2_O_2_ significantly accelerated the degradation of various organic pollutants and the detoxification of Cr (IV) through a reagent-free double-cathode electro-Fenton process (Figure 4c).

### 3.4. Effect of Surface Functional Groups or Substituent Groups

**Figure 5:**
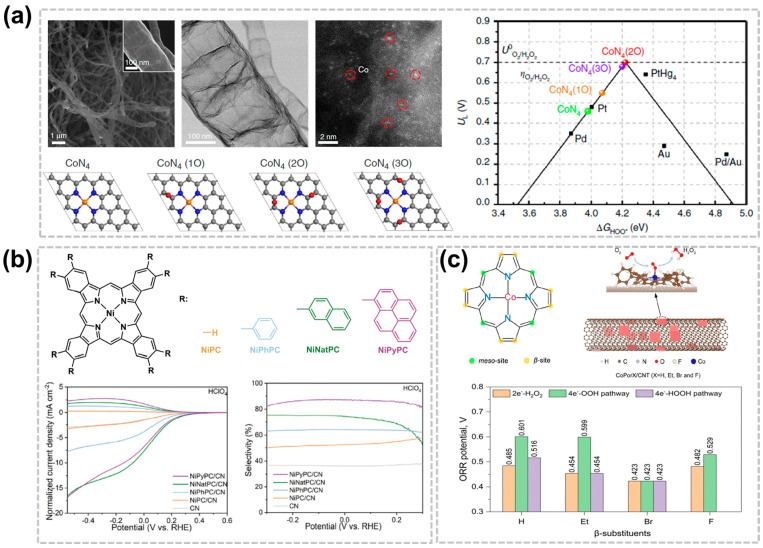
Effect in acidic H_2_O_2_ production of surface functional groups or substituent groups of C-SACs. (**a**) Microstructural analysis of the CoN@CNTs catalyst and DFT results of the various types of epoxy-modified Co-N_4_ sites [57]. Copyright 2020, Springer Nature. (**b**) Conjugated nickel phthalocyanine derivatives and their electrocatalytic LSV in 0.1 M HClO_4_ [59]. Copyright 2023, Wiley-VCH. (**c**) Schematic illustration of the preparation of HMC for H_2_O_2_ electrosynthesis from ORR [24]. Copyright 2023, Royal Society of Chemistry.

The performance of C-SACs for the production of the H_2_O_2_ would also be enhanced by adjusting the material composition or surface functional groups. The performance of appropriately surface-treated C-SACs is comparable to that of noble metal-based catalysts. For example, oxygen-containing functional groups can be formed on the surface of carbon materials such as carboxyl groups, C-O-C, and C-OH, thereby improving catalytic performance by increasing active sites. Zhang et al. prepared a Co and N co-doped carbon nanotubes (CoN@CNTs) composite by modulating the oxygen functional groups near the cobalt sites, exhibiting an excellent activity and selectivity of H_2_O_2_ production via the ORR in acidic solution [57]. Combined spectroscopic results and DFT demonstrated that the enhancement of H_2_O_2_ generation performance originates from the addition of epoxy groups near the Co-N_4_ centers, which has resulted in the transition of the electronic structure of the Co atoms. Furthermore, a custom H_2_O_2_ electrolyzer was developed with CoN@CNTs as the cathode catalyst which is capable of producing more than 0.1 wt% H_2_O_2_ within 30 min, and with the promising potential for electro-Fenton water treatment (Figure 5a). Compared with the complex surface of carbon-based materials, MOF catalysts with M-N_4_ or M-N_5_ structures can better understand the reaction mechanism by deciphering the structure-performance relationship of electrocatalysts through atomic-level manipulation [21,43,58]. Sun et al. synthesized nickel phthalocyanine derivatives with modified conjugation degrees and found them to be pH-universally effective electrocatalysts for H_2_O_2_ generation after heterogenization on nitrogen-decorated carbon with increased conjugation degrees leading to improved H_2_O_2_ selectivity [59]. The *OOH binding energy is optimized by the different functional groups that regulate the d-band center. The NiPyCN/CN demonstrates a high electrosynthesis activity for H_2_O_2_ production, achieving an 85% Faraday efficiency of H_2_O_2_ in an acidic medium (Figure 5b). Cobalt porphyrins with various meso-substituents have been widely studied, but ꞵ substituents have been studied little [60,61]. Liu et al. replaced all eight β-H atoms of a cobalt tetraphenyl porphyrin with electron-donating substituents, including Et, Br, and F, and constructed the corresponding hybrid metal–organic frameworks (HMCs) by using the carbon nanotube substrate [24]. The DFT analysis indicated that the electronic properties and catalytic activity of the Co active center on the carbon nanotube substrate can be effectively controlled by the β-site substituents. The CoPorF/CNT catalyst shows the highest performance with large TOFs of 85.1 and 3.51 s^−1^ for the 2e^−^-ORR in the alkaline and acidic environment, respectively. Additionally, the mass activity can reach 10.76 mol g_cat_^−1^ h^−1^ (Figure 5c).

## 4. Conclusions and Outlook

The synthesis of H_2_O_2_ by an electrocatalytic 2e^−^-ORR is a green, economical, safe, and efficient production method, which can be a promising alternative to the traditional anthraquinone process. This direct synthesis approach can achieve a decentralized and continuous production of H_2_O_2_ with great significance and industrial value, especially in an acidic environment. We considered C-SACs as great potential catalysts for the 2e^−^-ORR in acidic media due to their tunable electronic structure, low cost, and high stability. In this review, we exhibited a thorough understanding of the reaction mechanism of the 2e^−^-ORR, highlighted the recent advances of the C-SACs, and the regulation strategies of catalyst active sites, including metal active centers, nitrogen species, heteroatom doping, surface functional groups, and local microenvironments, providing the valuable ideas and reference methods for guiding the subsequent design of high-performance catalysts.

Although the C-SACs have made substantial progress for the 2e^−^ ORR in acidic conditions, there are many problems and challenges for the synthesis of H_2_O_2_ in practical applications with low-cost, high activity, selectivity, and stability. In order to further improve the catalytic performance of H_2_O_2_ and consider its practical industrial application, the following aspects should be considered:

(1) Constructing SACs with higher loading and stability. Enhancing the reactivity of active sites and increasing the number of active sites is critical for the design and preparation of more active C-SACs. However, the C-SACs tend to form clusters or even nanoparticles owing to the high surface energy as the load on the individual atoms increases, thus losing the advantages of single atoms. Improving single metal loading while retaining its configuration is the key challenge. Therefore, efficient synthetic strategies, such as ionic exchange, co-precipitation, impregnation, chemical reduction, and high-temperature pyrolysis can be utilized to adjust the morphology and electronic structures of the active site in catalysts. In addition, the current stability problem of C-SACs has always existed, usually for tens of hours, which is obviously not consistent with the long-term durability of the catalyst. Due to the accumulation of H_2_O_2_ concentration produced in the long-term test, it has a certain oxidation capacity, which leads to the destruction of the catalyst. At the same time, a high concentration of H_2_O_2_ promotes the further reduction of H_2_O_2_, which makes it difficult to improve the concentration further. Therefore, we need to design more efficient, high-capacity, and electrochemically stable C-SACs to meet their application under industrial conditions.

(2) Deeper understanding of the structure-activity relationship between active sites and properties of catalysts. At present, the design of the active sites in the catalysts with high-performance can be guided by strategies such as heterogeneous heteroatom doping, surface functionalization, defect engineering, and local microenvironment control of single atomic sites. However, the difference in the introduced catalytic active sites, the uncertainty of the active site location, and the effect of the association between the various active sites on the activity have not been clear. Therefore, it is still a great challenge to identify the effects of different active sites on catalytic performance and establish the effective structure-activity relationship between the active sites and catalytic performance. The stable coordination materials, such as MOF and covalent organic framework, can be used as precursors to combine with carbon materials to precisely adjust the electronic structure of the supporter in future research. Moreover, the structure-activity relationship between the active sites can be deeply explored with advanced spectroscopic characterization techniques to provide guidelines for the design of high-performance catalysts in the future.

(3) The challenges of industrial-grade production of H_2_O_2_. In order to further enhance the synthesis efficiency of H_2_O_2_ and satisfy the engineering needs of practical applications in industry, the design and development of catalysts should focus on low-cost, energy consumption, and high performance, ensuring stable operation for more than 100 h at more than 300 mA/cm^−2^. In recent years, transition metal carbon-based single-atom catalysts have demonstrated the excellent selectivity and production efficiency of industrial-grade current density in the flow cell. However, the long-term structural stability issue is unresolved, hindering their further commercial development. Therefore, improving the stability of catalysts for structure and activity will be the key point toward achieving the industrial and efficient synthesis of H_2_O_2_ in the future.
